# A retrospective study on the efficacy of integrating traditional Chinese medicine with Western medicine in the treatment of adult measles

**DOI:** 10.1186/s41065-025-00518-0

**Published:** 2025-07-26

**Authors:** Xiaoyan Liu, Zhongying Bao, Shuhong Duan, Jing Sun, Jie Zhang

**Affiliations:** Department of Infectious Diseases, Beijing Shijitan Hospital, Capital Medical University, No.10 of Tieyi Road, Yangfangdian, Haidian District, Beijing, 100038 China

**Keywords:** Adults, Antiviral drugs, Efficacy, Measles, Traditional chinese medicine (TCM)

## Abstract

**Objective:**

Measles is a highly contagious, potentially fatal disease, and using western medicine for treating this disease showed some limits including the long period of treatment and some side effects. Of note, traditional Chinese medicine (TCM) exhibited advantages of treating measles in using alone or combing with western medication. However, little information on the clinical study on adjunctive efficacy of TCM on measles. Therefore, the objective of this study is to observe the adjunctive efficacy of TCM in the treatment of adult measles.

**Methods:**

Ninety-one patients diagnosed with adult measles in our hospital were enrolled for this study. All of them underwent symptomatic treatment, nutritional support therapy, and ribavirin antiviral therapy. Loxoprofen was added to the treatment regime when the body temperature exceeded 38.5 °C. The patients were stratified into two groups based on the administration of TCM, comprising 49 individuals in the TCM group and 42 individuals in the control group. Patients in the TCM group voluntarily received the following TCM prescriptions: 15 g of *Lonicerae Japonica*, 15 g of *Forsythiae Fructus*, 15 g of *Arnebiae Radix*, 15 g of *Cicadae Periostracum*, 30 g of *Phragmitis Rhizoma*, 10 g of *Anemarrhenae Rhizoma*, 6 g of *Glycyrrhizae Radix et Rhizoma* and 30 g of *Gypsum Fibrosum* was administered to patients with high fever. Decoction: 1 dose daily for 5 days. The control group received treatment without the incorporation of TCM.

**Results:**

In comparison to the control group, patients in the TCM group experienced a quicker resolution of clinical symptoms (*P* < 0.05) and a lower complication rate (*P* < 0.05).

**Conclusion:**

The application of TCM in treating adult measles can shorten the duration of the disease, reduce the incidence of complications, and offers promise for wider clinical adoption and utilization.

## Introduction

Measles is an acute respiratory infectious disease caused by the measles virus. The primary clinical manifestations include fever, cough, runny nose, and other upper respiratory catarrhal symptoms, as well as conjunctivitis, Koplik’s spots in the oral mucosa, and a cutaneous maculopapular rash. The measles virus is transmitted through respiratory droplets, with the highest incidence occurring in winter and spring season.

Measles is a global epidemic infectious disease [1] that was once a leading cause of child mortality. It is highly contagious and prone to outbreaks and epidemics. Measles has the potential to become the third infectious disease to be eradicated through vaccination, following the global eradication of smallpox and the imminent eradication of polio. In China, the incidence of measles has significantly decreased since the widespread vaccination of infants and young children against measles in 1965, especially after it was included in the planned immunization implementation in 1978 [2]. According to data from the World Health Organization (WHO) and the Centers for Disease Control (CDC), in 2000, this highly contagious viral disease resulted in a total of 853,000 infections [3] and 535,000 deaths [4] worldwide. Between 2000 and 2018, global measles-related deaths decreased by 73% due to measles vaccination. However, measles remains prevalent in numerous developing countries, particularly in regions of Africa and Asia [5]. In 2018, over 140,000 people died from measles. The vast majority (over 95%) of deaths due to measles were in countries with low per capita incomes and inadequate health infrastructure [5]. The WHO-Western Pacific Region, which includes China, established a goal in 2005 to eradicate measles by 2020 [6]. However, the current progress indicates that the goal is not on track to be achieved.

In previous clinical practice, the traditional Chinese medicine (TCM) treatment plan prescribed by the famous medicine practitioner in our hospital have been applied for treating measles for many years. As a TCM empirical prescription, the herbal composition and dosage have been established through extensive clinical practice, performing beneficial therapeutic efficacy in clinical applications. Therefore, this study aimed to analyzed previous cases of measles in adults treated with TCM, and observe the efficacy of TCM in the treatment of adult measles. We have found that TCMs could shorten the duration of measles infection and decrease complications, ultimately achieving the objective of reducing severe illness and mortality.

## Data and methods

### General data of patients

This study was a retrospective cohort analysis involving the selection of 91 patients diagnosed with measles and treated in our department between January 1, 2000, and December 31, 2019. All patients were over 18 years old of age and exhibited typical symptoms of fever, respiratory catarrhal symptoms, and rash, as well as testing positive for measles IgM antibodies. All patients underwent antiviral therapy with ribavirin in addition to symptomatic treatment and nutritional support. Concurrent treatment with TCM was administered to 49 patients. Comprehensive medical records covering a three-week period subsequent to the initiation of symptomatic treatment were accessible for analysis. In the current study, it was observed that the time taken for clinical symptoms to improve in both groups of patients, including the duration of rash subsiding, the time taken for fever to subside, cough and sputum to cease, and symptoms such as photophobia, tearing, and runny nose to resolve. Meanwhile, acute complications of measles, such as laryngitis, pneumonia, myocarditis, and encephalitis, were observed. All patients had comprehensive medical records and records of auxiliary examinations conducted at least twice within 3 weeks of the onset of symptoms.

Compliance with the ethical standards of the hospital were strictly followed during the study and was reviewed by the hospital ethics committee. The study was approved by the Ethics Review Committee of Beijing Shijitan Hospital (No. IIT2024-025-001).

### Diagnostic criteria

The diagnostic criteria for measles were referenced from the *Diagnosis of Measles* (WS296-2017) [7]: with (or without) an epidemiologic history of measles, typical fever, respiratory catarrhal symptoms, and measles rash, along with the presence of positive IgM for measles (ELISA). It is important to rule out other diseases to make a clear diagnosis.

### Grouping criteria

Patients who received TCM treatment were assigned to the TCM group, while patients who did not receive TCM treatment were assigned to the control group. There were no gender or age differences between the TCM and the control group.

All patients have conducted the inspections on liver (Alanine transaminase [ALT]) function and cardiac enzymes (creatine kinase-MB [CK-MB]) pre-treatment. The patients in both control and TCM groups underwent symptomatic treatment, nutritional support therapy, and ribavirin antiviral therapy. When the body temperature exceeded 38.5 ℃, loxoprofen was administered to reduce fever, along with additional symptomatic treatment as needed. Both groups received a light diet and sufficient fluid intake during the treatment period. Efforts were made to keep the skin and mucous membranes clean and dry. Patients with pneumonic complications were prescribed oral levofloxacin 0.5 g once daily for 3 days.

Patients in the TCM group were given the following TCM prescription: 15 g of Lonicerae Japonica, 15 g of Forsythiae Fructus, 15 g of Arnebiae Radix, 15 g of Cicadae Periostracum, 30 g of Phragmitis Rhizoma, 10 g of Anemarrhenae Rhizoma, 6 g of Glycyrrhizae Radix et Rhizoma and 30 g of Gypsum Fibrosum were administered to patients with high fever. The ingredients were soaked in cold water for 1 h, and then boiled for 20 min. The resulting 200 ml suspension was divided into two portions and consumed while warm for 5 consecutive days.

The TCM prescription was not administered in the control group. Instead, they were provided symptomatic treatment, nutritional supportive therapy, and ribavirin antiviral therapy; loxoprofen was administered as needed when the body temperature exceeded 38.5 ℃.

### Exclusion criteria

Patients with chronic underlying conditions such as diabetes, acute or chronic organ insufficiency, a history of tumors, immune dysfunction, etc., were excluded from the study. Additionally, female patients who were pregnant or lactating were also excluded.

### Test method

The clinical cure criteria for measles are defined as the normalization of body temperature, complete resolution of the rash, disappearance of respiratory symptoms (including cough and rhinorrhea), and full recovery from any associated complications. If the patients’ ALT and CK-MB levels were within the normal range before treatment and their clinical observation indicators improved, these biochemical indicators would not be routinely retested.

### Statistical analysis

The data were statistically analyzed using SPSS 26.0 software. Normally distributed measured data are expressed as mean ± standard deviation (‾x ± s), and the independent sample *t*-test was used for comparison between groups. Non-normally distributed measured data are expressed as median (quartile) M (QL, QU), and the rank sum test was used. Count data are expressed as frequency and percentage (n %), and the chi-squared test or Fisher’s exact test was used to compare samples. *P* < 0.05 indicates a statistically significant difference, while *P* < 0.01 indicates a highly statistically significant difference.

## Results

The TCM group consisted of 49 individuals, including 28 males and 21 females, aged between 18 and 42 years, and with a mean age of 26.0 ± 6.9 years. The control group consisted of 42 individuals, including 24 males and 18 females, aged between 19 and 39 years, and with a mean age of 24.0 ± 5.1 years. There was no statistical difference between the two groups in terms of gender (male: 57% vs. 57%, *p* = 1.000) and age (26 ± 6.9 vs. 24 ± 5.1, *p* = 0.112, Table 1).

**Table 1:** The baseline information of adult patients with measles in both TCM group and the control group

	TCM Group (*n* = 49)	Control Group (*n* = 42)	t / χ^2^	*P*
Gender (M, %)	28 (57%)	24 (57%)	0.00	1.00
Age	26.0 ± 6.9	24.0 ± 5.1	1.60	0.11
IgM antibody positivity rate	49 (100%)	42 (100%)	0.00	1.00
ALT (U/L)	45.0 ± 10.0	40.2 ± 8.5	1.50	0.12
CK-MB (U/L)	25.3 ± 7.1	22.6 ± 6.4	0.21	1.10

*Note: ALT (Alanine transaminase), CK-MB (creatine kinase isoenzyme)

There was a statistically significant difference between the TCM and control groups in terms of the time required for symptoms to subside and the incidence of complications. The time required for symptoms to subside includes the time for fever reduction (2.2 ± 0.4 [TCM group] vs. 3.2 ± 0.3 [control group], *p* = 0.038), rash resolution (3.5 ± 0.7 [TCM group] vs. 4.1 ± 0.3 [control group], *p* = 0.028), cough remission (2.8 ± 0.3 [control group] vs. 4.2 ± 0.3 [control group], *p* = 0.041), photophobia relief (1.4 ± 0.6 [control group] vs. 2.3 ± 0.3 [control group], *p* = 0.047), tearing relief (1.7 ± 0.6 [control group] vs. 2.2 ± 0.4 [control group], *p* = 0.048), and nasal congestion and runny nose remission (1.9 ± 0.5 [control group] vs. 2.1 ± 0.3 [control group], *p* = 0.046). In the control group, five complications were observed, consisted of 3 cases of pneumonia and 2 cases of abnormal liver function, whereas no complications were noted in the TCM group. There was a statistically significant difference between the two groups (*p* < 0.019, Table 2).

**Table 2:** Comparison of the symptoms and complications of adult patients with measles in TCM group and control group

Symptom disappearance time (day)	TCM Group	Control Group	T values/ x^2^	*P* values
Fever	2.2 ± 0.4	3.2 ± 0.3	28.0211	0.038*
Rash subsided	3.5 ± 0.7	4.1 ± 0.3	17.1612	0.028*
Cough	2.8 ± 0.3	4.2 ± 0.3	27.1287	0.041*
Photophobia	1.4 ± 0.6	2.3 ± 0.3	10.1056	0.047*
Shed tears	1.7 ± 0.6	2.2 ± 0.4	11.1733	0.048*
Runny nose	1.9 ± 0.5	2.1 ± 0.3	5.8416	0.046*
Complications	0	5	5.499	0.019*

*P < 0.05

## Discussion

Measles is one of the most contagious diseases prevalent globally, shown by a R naught (R0) value of 12–18 [8, 9]. It has the potential to cause severe complications and even death. Measles can affect anyone, but it is most prevalent in children, and particularly those aged between 6 months and 5 years. It is one of the most widespread infectious diseases that contribute to child mortality [5]. As we all known, vaccines are the major tool in fighting against viruses in humans, which have played particularly important role in patients with abnormal immune status or tumors [10]. There are no specific antiviral medications for measles, and treatment is limited to managing symptoms.

Due to the widespread use of the measles vaccine, the global incidence of measles has significantly declined. The measles vaccine is the most effective, safe, and economical way to prevent measles. Prior to the introduction and widespread vaccination against measles in 1963, measles epidemics occurred approximately every two to three years and would cause an estimated 2.6 million deaths annually. Widespread vaccination against measles has significantly reduced the mortality rate resulting from measles in children [11]. In China, measles vaccination has been widely administered to infants and young children since 1965, and the measles vaccine was included in the free immunization program in 1978. This has led to a significant decline in the incidence of measles [2]. In 2001, the Measles Initiative was jointly launched by the American Red Cross, the CDC, the United Nations Foundation, United Nations International Children’s Emergency Fund, and the World Health Organization. The objectives of the Initiative are to eliminate deaths caused by measles in childhood, and the emergence of congenital rubella syndrome, reduce measles deaths by 95% by 2015, and achieve measles and rubella elimination in at least five WHO regions by 2020. However, the current program aimed at eradicating measles is clearly not on track, as occasional isolated mini-epidemics of measles continue to occur.

In 2017, the WHO announced that measles had been eliminated in the United Kingdom, which is defined as “the absence of circulating measles”. However, in 2018, around 900 cases of measles broke out in England. From 2022 to 2023, the number of measles cases in WHO Europe increased 45 times. In 2023, 40 number countries in the region reported about 42,200 measles cases, up from less than 1,000 in 2022 [12]. From October 2023 to January 2024, more than 300 cases of measles were reported in England alone [13]. Europe is facing a more alarming situation.

The United States has declared the indigenous eradication of measles outbreaks since 2000. However, the United States has witnessed resurgent measles outbreaks in recent years. In 2019, the Centers for disease control and prevention (CDC) documented 1,274 confirmed cases nationwide. According to the latest data from Johns Hopkins University in the United States, there have been at least 1,277 confirmed cases of measles reported in the United States in 2025, including three fatalities. Remarkably, the total number of cases in just the first half of the year has already surpassed the total for the entire year of 2019, making it the largest outbreak in three decades [14]. Similarly, the situation in Europe is equally alarming. According to data from the World Health Organization (WHO), the European region reported a total of 127,350 measles cases in 2024, marking the highest number since 1997 [15].

The onset of the COVID-19 pandemic in late 2019 has impacted the surveillance of measles cases and immunization efforts globally. The suspension of global immunization services and declining immunization rates has left millions of children vulnerable to preventable diseases such as measles [16].– [17] On November 17, 2023, the WHO released the report *Progress Towards Global Measles Elimination: 2000–2022* [18], noting that measles vaccination coverage has declined in recent years. In comparison to 2021, there was an 18% increase in measles cases in 2022 and a 43% increase in global deaths. In 2022, 37 countries experienced significant or devastating measles outbreaks. Measles outbreaks are on the rise, posing challenges to efforts to eradicate the disease. In 2021, the WHO reissued the *Measles and Rubella Strategic Framework: 2021–2030* [19], which outlines the goal of achieving and maintaining regional measles eradication by 2030. Hence, there is still a significant amount of work to be done in the battle against the measles virus.

The measles virus is classified as a member of the genus *Morbillivirus* in the Paramyxoviridae family. It is a single-stranded negative-sense RNA virus. Humans are the sole natural hosts of the measles virus. The measles virus typically manifests an average incubation period of approximately 10 days. A characteristic clinical symptom is the development of a rash across the entire body, typically occurring 3 to 4 days following the onset of fever [20]. Early symptoms encompass fever, cough, runny nose, conjunctival congestion, tearing, photophobia, and the appearance of Koplik’s spots in the oral mucosa on the 2nd to 3rd day of the illness. In typical cases, the rash appears on the 3rd or 4th day after the onset of fever and spreads sequentially from behind the ears, to the face, trunk, and limbs. The rash is characterized by red maculo-papular lesions. The skin between the rashes appears normal. The rash typically lasts for 3 to 6 days before subsiding and may spread to the palms of the hands and the soles of the feet. After reaching its peak, the body temperature begins to drop, and the rash then subsides in the order of appearance. Measles can also lead to severe complications in malnourished children and immunocompromised individuals such as those with HIV infection, cancer, and individuals undergoing immunosuppressive therapy, and as well as in pregnant women. These complications include blindness, encephalitis, severe diarrhea, ear infections, pneumonia, myocarditis, and the rare but serious subacute sclerosing panencephalitis. If a woman contracts measles during pregnancy, it can lead to premature birth and low birth weight of the infant. Patients presenting with myocarditis and encephalitis have a higher likelihood of developing multiple organ dysfunction syndrome (MODS) and a greater mortality rate [7, 8]. The incidence of measles in adults has increased in recent years. This trend may be related to the mobility and clustering of the population, as well as the decrease in antibody levels from the childhood measles vaccine and the weakening of its protective efficacy [11, 18]. When adults are infected with the measles virus, they are more likely to experience severe clinical symptoms and develop complications [21]. 

Measles is a self-limiting disease. There is no specific drug for the measles virus, hence the treatment primarily involves symptomatic and nutritional supportive therapy. The broad-spectrum antiviral drug ribavirin has some efficacy [22, 23]. Ribavirin belongs to the nucleoside class of antiviral drugs. Its specific mechanism of action is not completely clear, but it can inhibit the replication of respiratory syncytial virus, influenza virus, hepatitis A virus, adenovirus, measles virus, and other viruses [24]. Ribavirin theoretically plays a role in disrupting the replication process of the measles virus. However, its uncertain clinical efficacy and potential liver and kidney side effects, along with reproductive toxicity, have resulted in the absence of an effective drug targeting the measles virus in Western medicine.

Measles is classified as an exogenous fever in TCM. This ancient disease has been discussed, documented, and treated for thousands of years in the history of TCM. There is a wealth of valuable experience in the field of TCM in the pathogenesis, diagnosis, and treatment of measles. According to TCM, measles is classified as a “warm disease” and “positive toxicity.” The therapeutic approach focuses on facilitating skin eruption, eliminating toxins, and nourishing yin [24]. The combined use of several TCMs can disperse lung congestion, transform phlegm, and reduce fever [24].– [25] Positive clinical outcomes have been achieved through the use of TCM in treating measles, depending on the clinical expertise. The duration for relief from clinical symptoms and complication rate among the 49 patients in the TCM group who were treated with a combination of TCMs, showed statistically significant differences in comparison to those in the control group. This therapeutic approach resulted in good efficacy. In this prescription, *Lonicerae Japonica* [26] and *Forsythiae Fructus* [27] are the primary herbs (Monarch medicines) belonging to the category of herbs that clear heat and eliminate toxins, cool and release the exterior, and induce sweating. The main chemical components existed in *Lonicerae Japonica* and *Forsythiae Fructus* have been demonstrated to possess antiviral, anti-inflammatory, and antioxidant effects, for example, forsythoside A [28], chlorogenic acid [29], tannins [30] and so on. *Arnebiae Radix* [31, 32] is known for its medicinal properties, including treating blood heat [33], promoting blood circulation, detoxifying, and facilitating the eruption of rashes. It is particularly effective in treating conditions such as excessive blood heat and toxicity, as well as in cases where the eruption of measles or maculopapular rashes is not smooth. *Cicadae Periostracum* possessed the effects on wind-heat dispelling, throat-soothing, and rash-ventilating. *Phragmitis Rhizoma* is used as an adjuvant to clear lung heat and stomach fire, and has the effect of generating fluids and quenching thirst [34, 35]. *Anemarrhenae Rhizoma* has the effect of nourishing yin and moistening dryness. *Glycyrrhizae Radix et Rhizoma* has the effect of expelling phlegm and relieving cough, and regulating the efficacy of all medicines [36–38]. *Gypsum Fibrosum* has the effects of clearing heat and reducing fire, and removing vexation and quenching thirst when administered to patients with high fever. Previous reports have showed that *Gypsum Fibrosum* could performed antipyretic activity in yeast-induced rat model and the inorganic elements may be contributed to its bioactivity [39].

Complications prolong the course of treatment, resulting in increased medical expenses and heightened psychological stress for patients. In comparison to the control group, the 49 patients in the TCM group not only experienced quick relief from clinical symptoms but also did not develop acute complications of measles, such as pneumonia.

TCM treatment for measles can complement Western medicine by providing alternative approaches and possibilities for antiviral treatment, as well as reducing complications and mortality rates.

There are several limitations in this study. Firstly, it is a retrospective cohort study, and patients were not randomized but voluntarily chose to receive TCM treatment. This may have led to a better compliance to treatment in the TCM group, potentially introducing placebo effect and selective bias, for example, loss to follow-up bias. Additionally, the evaluation of the duration of the clinical symptom subsidence relied on self-assessment of the patient, perhaps leading to a memory bias, as well as the bias induced by the patients in subjective predisposition of the willingness of receiving TCM. Moreover, the study excluded individuals with diabetes, acute and chronic organ insufficiency, tumors, immune dysfunction, and other chronic underlying diseases, given that these conditions are recognized as posing a heightened risk of infection, increased susceptibility to infectious complications, and elevated mortality rates. However, there were some limits including the exist of age, the degree of education, base disease and compliance bias in present study. We will conduct prospective randomized double-blinds study in the further and collected the related information to decrease these biases.

## Conclusion

In this study, it was posited that the use of a combination TCM treatment for measles is feasible and effective and can rapidly and effectively reduce clinical symptoms. Aggressive treatment of patients with measles is essential for controlling the spread of the condition, reducing complications, and decreasing morbidity and mortality rates. To achieve the goal of eradicating measles, the most effective method is to increase the administration of the measles vaccine. Elevating the coverage of the measles vaccine and averting isolated outbreaks, as well as widespread epidemics, can contribute to a reduction in the incidence of measles in the foreseeable future. This aligns with the overarching global objective of the World Health Organization (WHO) to eradicate measles by the year 2030.

## Data Availability

The datasets used and/or analysed during the current study available from the corresponding author on reasonable request.

## Abbreviations

ELISA: Enzyme-linked immunosorbent assay
IgM: Immunoglobulin M
WHO: World Health Organization
CDC: Centers for Disease Control and Prevention
